# Empowerment Enabled by Information and Communications Technology and Intention to Sustain a Healthy Behavior: Survey of General Users

**DOI:** 10.2196/47103

**Published:** 2023-11-22

**Authors:** Ala Saleh Alluhaidan, Samir Chatterjee, David E Drew, Peter Ractham, Laddawan Kaewkitipong

**Affiliations:** 1 Department of Information Systems, College of Computer & Information Sciences Princess Nourah Bint Abdulrahman University Riyadh Saudi Arabia; 2 Center for Information Systems & Technology Claremont Graduate University Claremont, CA United States; 3 School of Educational Studies Claremont Graduate University Claremont, CA United States; 4 Center of Excellence in Operations and Information Management Thammasat Business School Thammasat University Bangkok Thailand

**Keywords:** empowerment, behavior change, information and communications technology, ICT, sustaining health behavior, long-term health behavior, mobile phone

## Abstract

**Background:**

Most people with chronic conditions fail to adhere to self-management behavioral guidelines. In the last 2 decades, several mobile health apps and IT-based systems have been designed and developed to help patients change and sustain their healthy behaviors. However, these systems often lead to short-term behavior change or adherence while the goal is to engage the population toward long-term behavior change.

**Objective:**

This study aims to contribute to the development of long-term health behavior changes or to help people sustain their healthy behavior. For this purpose, we built and tested a theoretical model that includes enablers of empowerment and an intention to sustain a healthy behavior when patients are assisted by information and communications technology.

**Methods:**

Structural equation modeling was used to analyze 427 survey returns collected from a diverse population of participants and patients. Notably, the model testing was performed for physical activity as a generally desirable healthy goal.

**Results:**

Message aligned with personal goals, familiarity with technology tools, high self-efficacy, social connection, and community support played a significant role (*P*<.001) in empowering individuals to maintain a healthy behavior. The feeling of being empowered exhibited a strong influence, with a path coefficient of 0.681 on an intention to sustain healthy behavior.

**Conclusions:**

The uniqueness of this model is its recognition of needs (ie, social connection, community support, and self-efficacy) to sustain a healthy behavior. Individuals are empowered when they are assisted by family and community, specifically when they possess the knowledge, skills, and self-awareness to ascertain and achieve their goals. This nascent theory explains what might lead to more sustainable behavior change and is meant to help designers build better apps that enable people to conduct self-care routines and sustain their behavior.

## Introduction

### Background

The US health care expenditure in 2020 was US $4.1 trillion, which accounted for 19.7% of the US gross domestic product [1]. In addition, the health care expenditure is expected to increase owing to societal aging [2]. Despite the rising expenditures, the United States has the highest rate of deaths amenable to health care among comparable countries [1]. Moreover, 6 in 10 Americans live with at least one chronic disease, such as heart disease, cancer, stroke, or diabetes, and 4 in 10 live with ≥2 of these chronic diseases. Chronic diseases are responsible for 7 out of every 10 deaths in the United States, killing more than 1.7 million Americans every year [3]. Negligence and health-risk behaviors are claimed to be among the leading causes of death in the United States [4]. Promoting healthy lifestyles and behaviors alone is not enough; healthy behavior should become an integral part of daily life [5]. Sustaining healthy behaviors such as regular physical exercise and healthy diet not only reduce serious chronic health conditions but also promote good health in the long term [5].

A need to focus more on sustaining healthy behavior has been highlighted by previous studies that showed that current health applications, including telemonitoring or home monitoring, have achieved only short-term success and adherence [6,7]. Sustaining healthy behavior can be achieved via patient or user empowerment, through which healthy behaviors can become regular habits [8]. Information and communications technology (ICT) tools, including mobile health (mHealth) applications, have been studied for their potential to support users in sustaining their health-protective behaviors by empowering them to keep track of their heart rate, blood glucose level, and exercise activities [9]. Prior research has found that empowerment is an important construct to improve the health of individuals with chronic conditions [9]. The World Health Organization [10] defines empowerment as “a process through which people gain greater control over decisions and actions affecting their health.” Therefore, it is important to understand how to foster empowerment so that people can decide and act intelligently to sustain their healthy behavior. However, prior research looked at empowerment as an outcome of the use of technology [11], and only a few of them examined the enablers of empowerment [12]. Therefore, this study attempts to contribute to the gaps by looking at empowerment as an antecedent to an intention to sustain healthy behavior (through the use of ICT, including mHealth technology) and investigating factors that contribute to the feeling of empowerment.

This research addresses the following research questions:

1. RQ1: What are the primary factors that affect feelings of empowerment and help toward sustainable behavior change?
2. RQ2: What is the effect of empowerment enabled by ICT on an intention to sustain a healthy behavior?

### Theory and Prior Work

One of the widely referenced theories in health informatics is the Integrated Theory of Health Behavior Change (ITHBC). According to this theory, knowledge and beliefs, self-regulation skills and abilities, and social facilitation are drivers for health behavior change [13]. To explain, an individual will engage in certain health behaviors if they have a positive attitude toward that behavior. Specifically, knowledge and beliefs affect behavior-specific self-efficacy. Self-regulation includes goal setting, self-monitoring, self-evaluating, and self-managing for physical, emotional, and cognitive reactions resulting from health behavior change. Social facilitation refers to social support and collaboration between families and health care providers. The goal of the ITHBC is to find ways to enhance a person’s engagement in behavior change and eventually improve self-management practices [13]. As our research goal was to explore factors that could reinforce the feeling of empowerment and thus enhance the intention to sustain a healthy behavior, ITHBC was applied as an overarching kernel theory for our research model.

Applying ITHBC to the context of ICT as an empowerment tool for health behavior change, we propose that the knowledge and beliefs should be adapted to having knowledge or being skillful in technology and the belief in self or self-efficacy. In addition, regarding the social connection in ITHBC, we propose that the influences of social connection and community support on empowerment should be explored. Social connection refers to a closer circle of friends and family to which a person relates, whereas community support refers to the larger circle where a person lives. Regarding self-management engagement, although ITHBC considers engagement as an important part of the path to health behavior change, it does not discuss the facilitation of such engagement. Thus, to better understand the facilitation of engagement, we refer to the approach of ICT empowerment via motivational messages and contents [14]. Empowerment can be reinforced through motivating messages that are aligned with a goal (eg, to be healthy) [15]. A review of techniques to increase engagement [16] pointed out that messages that align with personal goals and rewards are popular and successful techniques used for increasing mobile app user engagement. Therefore, we propose that messages that are aligned with personal goals and experientially rewarding content may help increase empowerment.

These factors could empower an individual and hence build an intention for a behavior. In the following sections, we present these factors and develop hypotheses on how they are related to empowerment and intention for sustaining healthy behavior.

### Hypotheses

#### Message Aligned With a Personal Goal

According to Abrahams et al [17], communication is an important factor that enhances patient empowerment. To achieve effective empowerment or self-management, including lifestyle modifications, it is crucial to motivate people [18]. An empowerment message should be highly relevant, match the recipient’s long-term goals, logically make sense and be achievable, make individuals feel good, and motivate an individual [19]. Therefore, this research assumes that “message alignment with a personal goal,” which also goes along the line with the motivation, would be more helpful in sustaining intended actions. Thus, we propose the following:

- H1: The more the messages are aligned with recipient’s goal, the more empowered they feel.

#### Experientially Rewarding Content

Experientially rewarding is a message that creates emotional connection as well as good, happy, and enthusiastic feelings that can motivate an individual. To increase consumer loyalty, stores implement experientially rewarding programs to attract new customers and retain existing customers [20]. Kolb and Kolb [21] stated that experiential reward learning enhanced the learning process more than plain instructions.

In the health care context, Liao et al [22] mentioned that establishing a clear reward mechanism could foster active engagement and empowerment. Thus, we predict that experientially rewarding content can help in seeding more motivation and empowerment. Therefore, we propose the following:

- H2: The more experientially rewarding content an individual is exposed to, the more empowered they feel.

#### Familiarity With Technology Tools

Familiarity with technology tools is a factor in increasing self-efficacy and ultimately empowering an individual [23]. According to Chen [24], individuals who are confident in their technology skills are more motivated and have more experience, which would lead to greater self-efficacy [24]. Thus, we predict the following:

- H3: The higher familiarity with technology tools an individual has, the higher perceived self-efficacy they feel.

#### Self-Efficacy

Self-efficacy is an individual’s belief in their capacity to execute behaviors. A theoretical and empirical literature review [25] emphasizes the role of self-efficacy and its significance in the health care community. Self-efficacy is relevant to the development of the ability to engage in and sustain positive health behaviors [26], whereas empowerment is about gaining control over health decisions [10]. According to Davies et al [27], self-efficacy and empowerment are different and not interchangeable concepts; they could be associated with each other in fostering healthy behavior. Therefore, we propose the following:

- H4: The higher perceived self-efficacy an individual has, the more empowered they will be.

#### Social Connection

According to the social support theory, social support system comprises family, friends, coworkers, and others who are socially connected [28]. It offers the members a feeling of belonging, security, and a greater sense of self-worth, and helps mediate and buffer stress [29,30]. More importantly, social support provides members with enhanced recovery and better compliance [31].

Zimmerman [32], who stated that empowerment is expressed at the psychological level, theorized that empowerment, from a psychological perspective, is maneuvered throughout interpersonal, interactional, and behavioral components. Accordingly, social connection (interpersonal and interactional connections with friends and family) could help increase the feeling of being empowered; thus, we propose the following:

- H5. The more socially connected an individual is, the more empowered they feel.

#### Community Support

On the basis of the self-determination theory, multiple constructs—autonomy, competence, and relatedness—are considered when explaining a behavior. Relatedness is the desire to feel connected to others and it can be seen in community support. Community-based interventions to increase physical activity, such as the percentage of people starting exercise programs and the frequency of physical activity, have proved to be more effective [33,34]. Community empowerment initiatives can help improve people’s health and can take many forms such as health promotions, workshops, healing groups, and drug prevention programs [35]. Therefore, community support is essential for any health intervention that aims for behavior sustainability. Previous research [36] has found that community support leads to greater empowerment and better quality of life.

Therefore, we propose the following:

- H6: The more community support an individual has, the more empowered they feel.

#### Feeling Empowered and an Intention for Action

On the basis of the Kanter’s [37] theory, an individual’s sense of empowerment related to self-determination, and self-determination, an individual’s belief on their ability to make their own choices, is a predecessor to an intention to act.

Previous research [38] found that patient empowerment was positively related to the intention of patients to sustain their engagement with web-based health infomediaries, whose platform enables exchanges of health information. Atak et al [39] found a positive relationship between patient empowerment and long-term health outcomes. On the basis of these findings, we propose the following hypothesis:

- H7: The more empowered an individual is, the more they intend on sustaining a healthy behavior.

**Figure 1:**
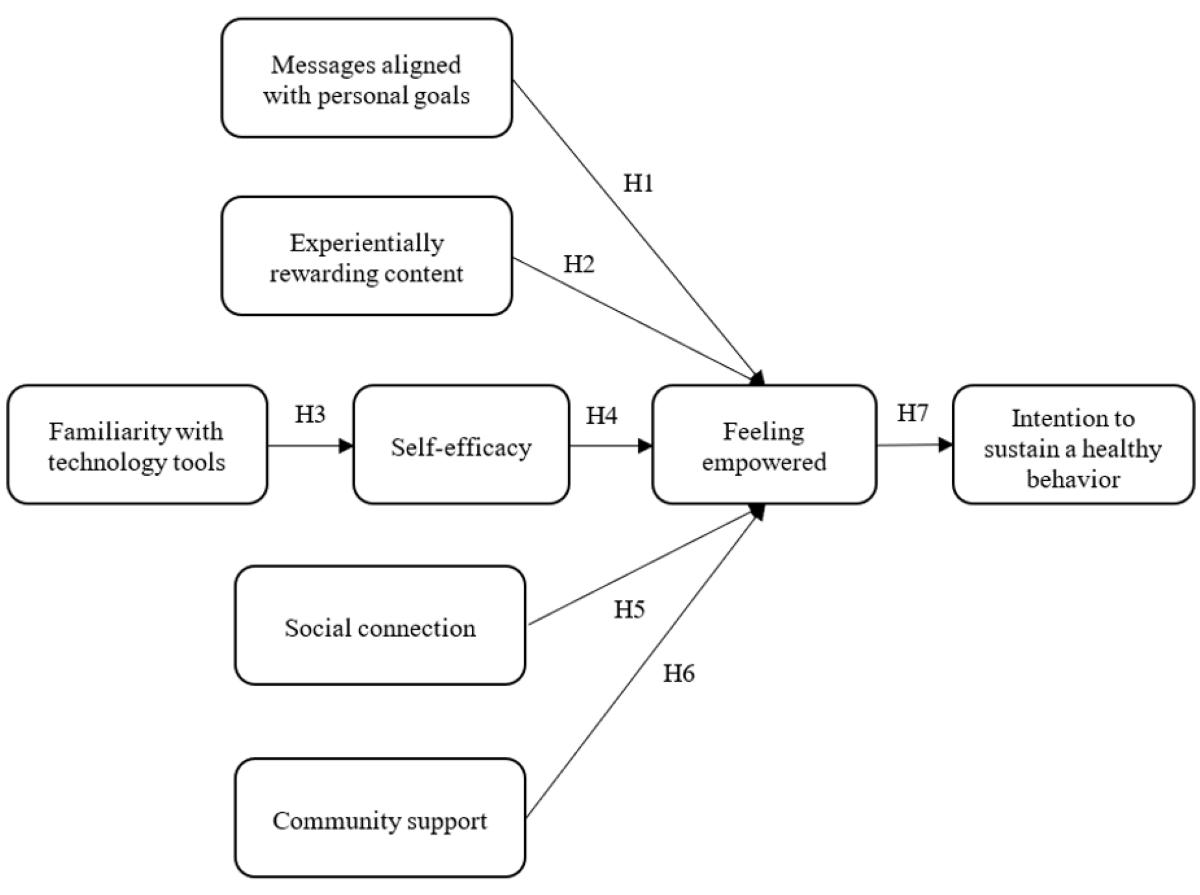
Research model.

From the above discussion, the research model is drawn in Figure 1. Arrow lines represent hypothesized causal paths from one variable to another.

## Methods

### Research Approach and Data Collection

This study used a quantitative survey approach to test the proposed theoretical model of empowerment and an intention to sustain healthy behavior. The authors surveyed English speakers aged >21 years. First, the survey was distributed via the mailing lists of 5 universities. Overall, 174 respondents completed the survey. This set of data was analyzed and published as a proceedings paper. Later, we complemented the number and expanded it to include samples that were not university students (for better distribution of samples and thus better generalization) with respondents from Amazon Mechanical Turk. Eventually, we collected 427 completed survey responses out of 458 (response rate≈93%).

### Construction of Variables and Measurement

#### Overview

The survey included questions about demographic information and questions for each measuring item. Additional instruction, asking respondents to assume that their personal goal is to be healthy and to exercise regularly, was specifically added to questions of “messages aligned with personal goals” construct. A total of 8 variables were included in the research model. Measure items for each variable were adapted from previous studies or relevant theories; a 5-point Likert scale was used for each measure item.

#### Message Aligned With a Personal Goal

According to Chatterjee et al [19], empowerment messages should be aligned with personal goals and experientially rewarding. We used 2 components, namely disease or health state and social network from [19] to develop empowerment messages that are aligned with personal goals. As each respondent may have different personal health-related goals, we adopted the more common goals (for those who are concerned about their health), which are to exercise regularly and to keep healthy. As mentioned earlier, respondents were asked to assume that their personal health-related goals were to keep healthy and to exercise regularly. Then, they will rate each message whether they find it aligned with the assumed personal goals.

#### Experientially Rewarding Content

According to Woolley and Fishbach [40], to assess rewards for pursuing one’s resolution (to regularly exercise in our case) is to measure happiness, enjoyment, and positive experience. However, as we did not intend to gauge the level of happiness, but we were interested in what kind of content or event that would make people feel experientially rewarding, so we applied internal (self-image or personality) and external (family support and socioeconomic status) facilitators to exercising [41] to develop events that, when happened, make a person feel good and happy. By asking a respondent about what event would make them feel good and happy, we expect to investigate what are rewarding experiences relevant to healthy behavior.

#### Familiarity With Technology Tools

Measure items for this construct were adapted from technological self-efficacy [42] and attitudes toward technology [43]. The items focused on capability to use, comfort with, and frequent use of technological tools. We use the term technology in general to refer to smartphones, internet, computers, televisions, and wearable devices (such as Fitbit).

#### Self-Efficacy

Measure items developed by Chen et al [44] were adopted. Self-efficacy is a well-known and widely used construct in studies relating to behavioral intention. It refers to an individual’s belief in his or her capacity to execute behaviors, for example, confidence in overcoming challenges and achieving intended tasks or goals.

#### Social Connection

According to Douglas [45], social connection is a sense of connection one feels or has with their family and friends. It is important to personal well-being. Examples of social connection expressions listed in the study by Douglas [45] were adapted as measure items for social connection.

#### Community Support

The measures were adapted from the Perceived Community Support Questionnaire by Herrero and Gracia [46]. According to Herrero and Gracia [46], community support can be divided into 3 dimensions, namely community integration, community participation, and community organizations. In our study, we adopted the 5-item scale that measures the degree of support a person perceives from his or her community, for example, “I would find someone to listen to me when I feel down.” and “I could find people that would help me feel better.”

#### Feeling Empowered

If a person is empowered, he or she can make effective choices [47]. Attributes of empowerment and a scale to measure empowerment [48] were adapted to build a list of questionnaire items for feeling empowered.

#### Intention to Sustain Healthy Behavior

We adapted the measure items of continuance intention [49], as it conveys the meaning of long-term behavior, and combined it with the 3 key healthy behaviors and mental health [50]. Examples of intention to sustain healthy behavior are intention to continue to exercise, eat healthy food, sleep well, manage stress, and maintain a work-life balance.

For common method bias, we eliminated item ambiguity by asking different people to read and explain their understanding. The survey was evaluated first by 8 individuals within academia before it was distributed. Details of measure items for each construct are presented in Table 1.

**Table 1:** Theoretical construct items.

Construct	Definition	Item code	Item questions
MA^a^ with personal goals	All messages (text) are in line with the participant’s goal (good health and regular exercise) toward certain behavior	MA1	You should eat ≥5 servings of fruits and vegetables (combined) daily
MA with personal goals	All messages (text) are in line with the participant’s goal (good health and regular exercise) toward certain behavior	MA2	You should eat foods low in fat
MA with personal goals	All messages (text) are in line with the participant’s goal (good health and regular exercise) toward certain behavior	MA3	Try getting 8 hours of sleep a day to keep stress away
MA with personal goals	All messages (text) are in line with the participant’s goal (good health and regular exercise) toward certain behavior	MA4	Drink at least 5 glasses of water a day which reduces the risk for heart attack and stroke by 41% in women and 54% in men
MA with personal goals	All messages (text) are in line with the participant’s goal (good health and regular exercise) toward certain behavior	MA5	By being physically active, you will lead a healthy and long-lasting life
MA with personal goals	All messages (text) are in line with the participant’s goal (good health and regular exercise) toward certain behavior	MA6	Smoking and excessive drinking is fine
ER^b^	These events make the participants feel good and happy	ER1	Spending time with my family gives me motivation to exercise
ER	These events make the participants feel good and happy	ER2	Getting recognized for my accomplishments
ER	These events make the participants feel good and happy	ER3	Receiving some award when I achieve my physical exercise goal
ER	These events make the participants feel good and happy	ER4	If you exercise, you will look more attractive
ER	These events make the participants feel good and happy	ER5	If you exercise, your insurance will go down
TT^c^	We use the term technology in general to refer to smart phones, internet, computers, televisions, and wearable devices (such as Fitbit)	TT1	I am comfortable using technology
TT	We use the term technology in general to refer to smart phones, internet, computers, televisions, and wearable devices (such as Fitbit)	TT2	I feel more capable with my smartphone
TT	We use the term technology in general to refer to smart phones, internet, computers, televisions, and wearable devices (such as Fitbit)	TT3	I can accomplish most of my tasks using computers, internet, and technology
TT	We use the term technology in general to refer to smart phones, internet, computers, televisions, and wearable devices (such as Fitbit)	TT4	I often use the internet to look for solutions to problems
TT	We use the term technology in general to refer to smart phones, internet, computers, televisions, and wearable devices (such as Fitbit)	TT5	I feel powerless without technology
TT	We use the term technology in general to refer to smart phones, internet, computers, televisions, and wearable devices (such as Fitbit)	TT6	I have used technology to motivate me to do physical exercise
TT	We use the term technology in general to refer to smart phones, internet, computers, televisions, and wearable devices (such as Fitbit)	TT7	I do not see the need for technology tools
SE^d^	Refers to an individual’s belief in his or her capacity to execute behaviors	SE1	I will be able to achieve most of the goals I set for myself
SE	Refers to an individual’s belief in his or her capacity to execute behaviors	SE2	When facing difficult tasks, I am certain I will succeed
SE	Refers to an individual’s belief in his or her capacity to execute behaviors	SE3	I believe I can succeed at most tasks to which I set my mind
SE	Refers to an individual’s belief in his or her capacity to execute behaviors	SE4	I will be able to successfully overcome many challenges
SE	Refers to an individual’s belief in his or her capacity to execute behaviors	SE5	I am confident I can manage well on many different tasks
SE	Refers to an individual’s belief in his or her capacity to execute behaviors	SE6	Compared with other people, I can do most tasks very well
SE	Refers to an individual’s belief in his or her capacity to execute behaviors	SE7	Even when things are tough, I can manage quite well
SC^e^	The number of family, friends, and social acquaintances that the participant connects to	SC1	I have a friend or family member who encourages me to accomplish my goal
SC	The number of family, friends, and social acquaintances that the participant connects to	SC2	I often feel very lonely
SC	The number of family, friends, and social acquaintances that the participant connects to	SC3	My family members are always there to help and support me
SC	The number of family, friends, and social acquaintances that the participant connects to	SC4	In the past month, it has been easy to relate to my friends and family
CS^f^	Community support, which implies help from friends, neighborhood, churches, and other social environment	CS1	My community helps me to be cheerful
CS	Community support, which implies help from friends, neighborhood, churches, and other social environment	CS2	In my community, I would find a source of satisfaction for myself
CS	Community support, which implies help from friends, neighborhood, churches, and other social environment	CS3	In my community, I would find someone to listen to me when I feel down
CS	Community support, which implies help from friends, neighborhood, churches, and other social environment	CS4	In my community, I could find people that would help me feel better
CS	Community support, which implies help from friends, neighborhood, churches, and other social environment	CS5	In my community, I would relax and easily forget my problems
CS	Community support, which implies help from friends, neighborhood, churches, and other social environment	CS6	In my community, I take part in activities
CS	Community support, which implies help from friends, neighborhood, churches, and other social environment	CS7	I respond to calls for support in my community
FE^g^	Having a positive attitude toward life and feeling more capable to achieve positive results	FE1	I have a positive attitude toward life
FE	Having a positive attitude toward life and feeling more capable to achieve positive results	FE2	Having access to information and resources enables me to take proper informed decisions
FE	Having a positive attitude toward life and feeling more capable to achieve positive results	FE3	I go out of my way to help others
FE	Having a positive attitude toward life and feeling more capable to achieve positive results	FE4	I feel the ability to change other’s perceptions by democratic means
FE	Having a positive attitude toward life and feeling more capable to achieve positive results	FE5	I have a positive self-image and I can overcome stigma
ISHB^h^	Forming a plan to maintain the behavior for a long time	ISHB1	I intend to continue to exercise
ISHB	Forming a plan to maintain the behavior for a long time	ISHB2	I intend to eat healthy from now on
ISHB	Forming a plan to maintain the behavior for a long time	ISHB3	I intend to keep a work-life balance going forward
ISHB	Forming a plan to maintain the behavior for a long time	ISHB4	I intend to sleep well and manage my stress from now on
ISHB	Forming a plan to maintain the behavior for a long time	ISHB5	From now on I will continue to remain healthy
ISHB	Forming a plan to maintain the behavior for a long time	ISHB6	Technology tools help me better manage my exercise routines
ISHB	Forming a plan to maintain the behavior for a long time	ISHB7	With or without support, I intend to stay physically fit

^a^MA: messages aligned.

^b^ER: experiential rewards.

^c^TT: technological tools.

^d^SE: general self-efficacy.

^e^SC: social connection.

^f^CS: community support.

^g^FE: feeling empowered.

^h^ISHB: intentions to sustain a health behavior.

### Statistical Analysis

We used structural equation modeling to determine whether and to what extent messages aligned with personal goals, experientially rewarding content, general self-efficacy, which is subsequently affected by experience in using technological tools, social connection, and community support affect the intention to sustain a healthy behavior by building empowerment feelings. We analyzed the collected data using AMOS (version 23.0; IBM Corp) and SPSS (version 23.0; IBM Corp).

To measure the internal consistency reliability, Cronbach α and composite reliability were calculated [51]. Discriminant validity was checked by determining that the square root of average variance extracted (AVE) for each construct is greater than the correlation between that construct and others [52].

The root-mean-square error of approximation (RMSEA), the comparative fit index (CFI), and the chi-square test of model fit were used to evaluate a good fit of the research model [53]. RMSEA indicates the extent to which the hypothesized model is from a perfect model, whereas CFI indicates the fit of the hypothesized model with that of a baseline model [54]. In addition to the fit indices, we looked at the standardized path coefficient to determine an effect of the change of one variable on another variable.

The hypothesis was accepted or rejected based on *P* value, path coefficient, and *t* value. To accept the hypothesis, the *P* value should be <.05, the path coefficient (a value ranging from −1 to 1) absolute value should be >0.3, indicating a moderate or strong (if the path coefficient is higher) relation between the 2 factors, and the *t*-statistic should be >2.0, indicating the significance of the coefficient.

### Ethical Considerations

Ethical approval was obtained from the institutional review board at Claremont Graduate University (#2656). Participation in the survey was on a complete voluntary basis. The first page of the survey contained a consent form, which informed a respondent that no personal data would be collected, and thus their answers would be completely anonymous. In addition, respondents were informed that they could quit the survey at any point if they changed their minds and no longer wanted to participate. As the survey was distributed on websites, the respondents were able to answer all the questions at their convenience and privacy without potential influences that may occur in the presence of the researchers.

## Results

### Respondent Descriptive Statistics

Table 2 shows the overall respondent profiles. Of 427 respondents, 353 (82.7%) were aged between 21 and 39 years, 244 (57.1%) were women, 390 (91.3%) respondents considered themselves having good or very good or excellent health, and 249 (58.3%) respondents exercised regularly.

**Table 2:** Demographic characteristics of the respondents (N=427).

Classification			Respondents, n (%)
**Ethnicity**			
	White	163 (38.2)	
	Hispanic or Latino	49 (11.5)	
	Black or African American	24 (5.6)	
	Native American or American Indian	5 (1.2)	
	Asian or Pacific Islander	127 (29.7)	
	Other	59 (13.8)	
**Age (years)**			
	<30	221 (51.8)	
	30-39	132 (30.9)	
	40-49	41 (9.6)	
	50-59	23 (5.4)	
	≥60	10 (2.3)	
**Gender**			
	Men	183 (42.9)	
	Women	244 (57.1)	
**Marital status**			
	Single	251 (58.8)	
	Married	153 (35.8)	
	Separated	5 (1.2)	
	Divorced	17 (4)	
	Widowed	1 (0.2)	
**Education**			
	Less than high school	2 (0.5)	
	High school	18 (4.2)	
	College	205 (48)	
	Master or doctorate	202 (47.3)	
**Employment**			
	Self-employed	29 (6.8)	
	Employed	210 (49.2)	
	Retired	8 (1.9)	
	Student	161 (37.7)	
	Unemployed	19 (4.4)	
**Annual household income (US $)**			
	<24,000	152 (35.6)	
	25,000-49,000	103 (24.1)	
	50,000-999,000	102 (23.9)	
	≥100,000	70 (16.4)	
**Family members in the house**			
	Just me	102 (23.9)	
	Me and 1-2 members	151 (35.4)	
	Me and 3-4 members	136 (31.9)	
	Me and 5-6 members	27 (6.3)	
	Me and 7 or more members	11 (2.6)	
**Having a chronic disease**			
	Yes	86 (20.1)	
	No	341 (79.9)	
**Health assessment**			
	Excellent	75 (17.6)	
	Very good	170 (39.8)	
	Good	145 (34)	
	Fair	33 (7.7)	
	Poor	4 (0.9)	
**Exercise regularly**			
	Yes	249 (58.3)	
	No	178 (41.7)	
**Belief in exercise**			
	Strongly disagree	22 (5.2)	
	Disagree	1 (2)	
	Neutral	10 (2.3)	
	Agree	107 (25.1)	
	Strongly agree	287 (67.2)	

### Reliability and Validity of Constructs

Cronbach α (internal consistency) was used as an (lower bound) estimate of the reliability and the accepted values of α ranged from .7 to .95. All the constructs met this condition and the Cronbach α values were >.7. The composite reliability of each construct was calculated to check internal consistency validity. All constructs appeared to have high composite reliability values >0.6, which is considered acceptable [55]. Table 3 shows Cronbach α and composite reliability of each construct.

**Table 3:** Reliability and validity of factors.

Factor	Items	Cronbach α (N=427)	Composite reliability
MA^a^	MA∼4	.742	0.75
ER^b^	ER∼2	.772	0.786
TT^c^	TT∼4	.808	0.79
SE^d^	SE∼7	.841	0.833
SC^e^	SC∼3	.724	0.699
CS^f^	CS∼6	.894	0.74
FE^g^	FE∼5	.77	0.771
ISHB^h^	ISHB∼6	.836	0.885

^a^MA: messages aligned with personal goals.

^b^ER: experiential rewards.

^c^TT: technological tools.

^d^SE: self-efficacy.

^e^SC: social connection.

^f^CS: community support.

^g^FE: feeling empowered.

^h^ISHB: intentions to sustain a health behavior.

The square root of AVE (diagonal elements in Table 4) was calculated to check for discriminant validity. If the square root of the AVE values is higher than the correlation coefficient, high discriminant validity is achieved. In this research, with N=427, this condition is satisfied for all except for “feeling empowered” (FE). With N=427, FE has the correlation 0.676 with “intention to sustain a health behavior,” which is higher than the square root of AVE, 0.636.

**Table 4:** Discriminant validity test result.

	MA^a^	ER^b^	TT^c^	SE^d^	SC^e^	CS^f^	FE^g^	ISHB^h^
MA	*0.658* ^i^	N/A^j^	N/A	N/A	N/A	N/A	N/A	N/A
ER	0.068	*0.805*	N/A	N/A	N/A	N/A	N/A	N/A
TT	0.452	0.192	*0.697*	N/A	N/A	N/A	N/A	N/A
SE	0.362	0.113	0.399	*0.59*	N/A	N/A	N/A	N/A
SC	0.253	0.234	0.260	0.295	*0.68*	N/A	N/A	N/A
CS	0.256	0.140	0.271	0.378	0.490	*0.64*	N/A	N/A
FE	0.424	0.144	0.466	0.478	0.544	0.607	*0.636*	N/A
ISHB	0.512	0.135	0.372	0.472	0.355	0.489	0.676	*0.751*

^a^MA: messages aligned with personal goals.

^b^ER: experiential rewards.

^c^TT: technological tools.

^d^SE: self-efficacy.

^e^SC: social connection.

^f^CS: community support.

^g^FE: feeling empowered.

^h^ISHB: intentions to sustain a health behavior.

^i^The italicized values are the square root of average variance extracted values.

^j^N/A: not applicable.

### The Fitness Test of the Model

The model fit indices of our research model were found acceptable, with RMSEA=0.055 and CFI=0.87. In addition, Medsker et al [56] introduced the notion of chi-square and df as an index, treating ratios between 2 and 5 as indicating a good fit. The model displayed a reasonable fit with the data *χ*^2^/df=2.298.

### Structural Equation Modeling Analysis Results

**Figure 2:**
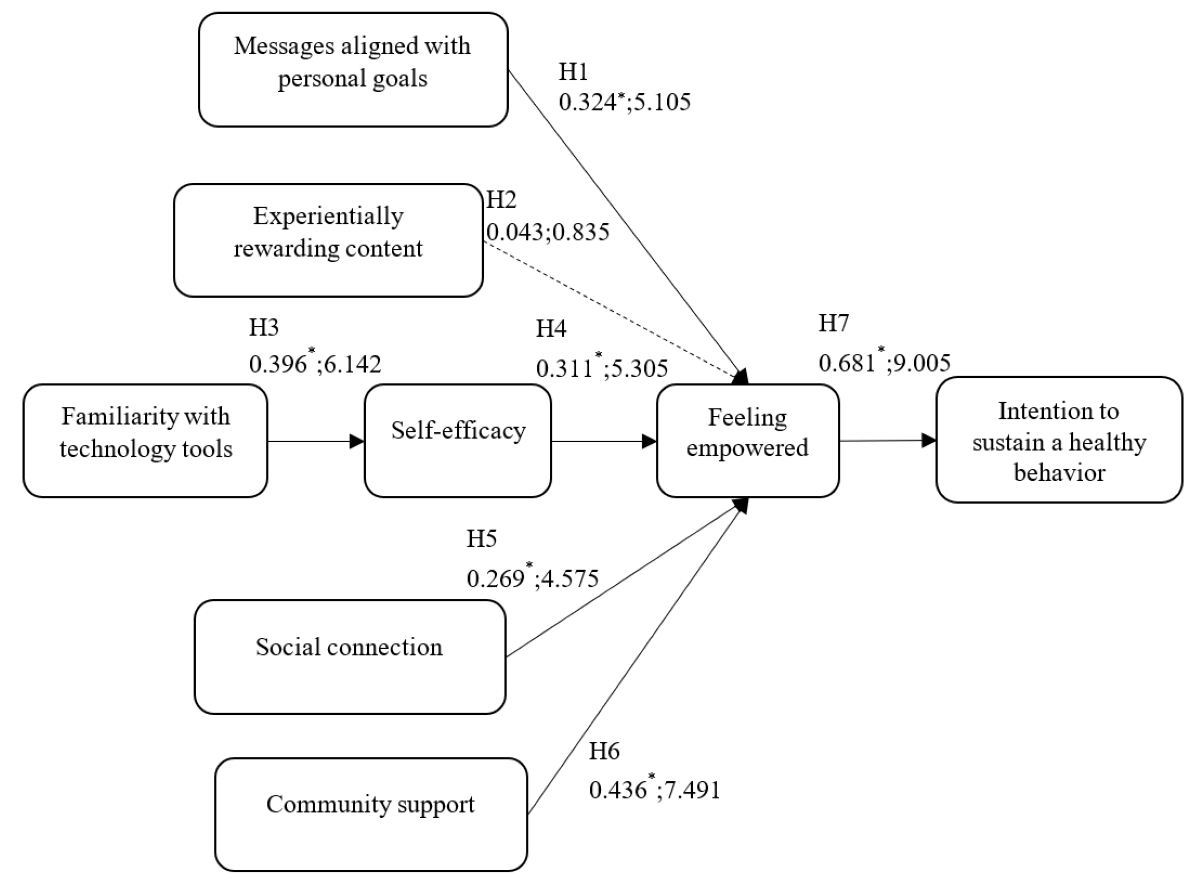
Path analysis results in the format “path coefficient; *t*-values” are reported. *Significant level at *P*<.001.

The path coefficient and *t* value are reported below, and the results are shown in Figure 2.

1. H1: The path coefficient of hypothesis 1 is 0.324, and the *t* value is 5.105; therefore, this hypothesis is supported. This means that the “message aligned with personal goals” is confirmed to be a supportive factor to empower individuals. Thus, we can say that the more aligned the messages with a person’s goals, the more empowered they feel.
2. H2: Hypothesis 2 is not supported. The path coefficient of hypothesis 2 was 0.043, and the *t* value was 0.835. Consequently, this hypothesis was rejected. Contrary to our expectations, the relationship between experiential rewards and feeling empowered was not significant. In other words, experiential rewards did not contribute to empowering individuals to maintain a healthy behavior.
3. H3: The path coefficient of hypothesis 3 is 0.396, and the *t* value is 6.142; thus, this hypothesis is supported. This result means that the more the individuals are familiar with technology tools, such as using smartphones and internet for performing daily tasks, the higher self-efficacy they possess.
4. H4: The path coefficient of hypothesis 4 is 0.311, and the *t* value is 5.305; therefore, this hypothesis is also supported. This result means that the higher the self-efficacy an individual possesses, the more empowered they feel.
5. H5: Hypothesis 5 is supported because the path coefficient is 0.269 and the *t* value is 4.575. The results can be interpreted as the more support an individual gain from family and friends who are connected to, the more empowered they feel.
6. H6: The path coefficient and *t* value are 0.436 and 7.491, respectively, and this hypothesis is supported. The results indicate that community support has a great influence on individuals and can improve their feelings of empowerment.
7. H7: The path coefficient and *t* value are 0.681 and 9.005, respectively, and the hypothesis is supported. The results indicate that the more an individual feels empowered, the more they develop an intention to sustain a healthy behavior.

## Discussion

### Principal Findings

The research found that message aligned with personal goals, self-efficacy, social connection, and community support positively affect an individual’s feeling of empowerment, which in turn affects their intention to sustain a healthy behavior. This result sheds light on the benefits of motivation and empowerment because the cost of noncompliance (not adhering to healthy behavior recommended by doctors and physicians) can include hospitalization and worsening of a health condition.

The findings are in line with previous studies (eg, [57-59]) that found self-efficacy, social life, and community support to be either directly or indirectly related to behavior change. In addition, this study found that familiarity with technological tools could enhance individuals’ perception of self-efficacy. Therefore, to encourage individuals to maintain their healthy behavior, ICT should be used as an empowering tool to enhance health outcomes [59]; however, it is important to note that the technology *per se* cannot effectively foster behavior change. Our research highlights that social factors, namely social connection and community support, are also important factors influencing feeling of empowerment. In addition, messages or contents that are conveyed on mHealth application are also found to have a positive influence on an intention to sustain healthy behavior, if the messages are personalized to align with users’ personal goals. Personal goals can be varied. Our measurement items for messages aligned with personal goal assumed a goal to exercise regularly for the respondents. The hypothesis result thus implies that if a person’s personal goal is to exercise regularly and the messages are designed to be relevant to regular exercises, the messages could positively affect the person’s feeling of empowerment.

Surprisingly, we found that experientially rewarding content had no impact on individuals’ feeling of empowerment. This also contradicts prior studies (eg, [40,60]), which stated that rewards or feeling rewarded contribute to persistence in long-term goals or behavior change. The concept of rewarding is also widely used in gamification and has been proven to be helpful in promoting the use of mHealth apps [61,62]. Thus, it is possible that experientially rewarding may not directly relate the feeling of empowerment, but may relate to the intention to adopt or maintain a particular behavior. Future studies should investigate this relationship.

### Theoretical Implications

This study makes a key theoretical contribution to a gap in theorizing how we empower citizens using ICT. We build on the ITHBC theory [13] as well as previous trial and empowerment messages, which were detailed as a model on in the study by Alluhaidan et al [14] and Chatterjee et al [19]. The factors introduced in our theoretical model and those obtained from the literature could be used for future research in the area of ICT empowerment and sustaining healthy behaviors. This research also contributes guidelines for how to construct items for a latent factor within the information systems domain, such as “messages aligned with personal goals.”

In addition, although the role of ICT on empowering and enhancing health behavior has been highlighted in previous literature [8,11], this research moved one step forward to focus on an intention to sustaining a healthy behavior, rather than just changes of behavior. This is important as an adoption of health technology was just an initial step that may not lead to health behavior improvement.

Finally, prior literature has looked at empowerment as a process or outcome [11], but only a few studies have examined the enablers of empowerment [12]. Therefore, our research adds to this gap by highlighting factors that could lead to an individual’s feeling of empowerment and intention to sustain a healthy behavior.

### Practical Implications

This study has several important implications. First, designers of ICT-enabled apps and tools should focus not only on designing features and functionalities but also on the messages used in the apps. The messages should be aligned with the user’s goals or purposes of using the apps. In addition, designers of the apps may consider adding features that would foster social connection and community support, such as allowing users to add other users into their circle and giving another user encouragement via star or gift sending. Such features could enhance the perception of community support and social connection, which in turn would lead to feeling of empowerment.

As an individual who wants to sustain a healthy behavior, one may seek support from their community or peers and stay connected with family and friends. These social factors would motivate a person to maintain positive health behavior. In addition, an individual may try to familiarize themselves with technology tools; this would help them become more confident and feel more competent to control or overcome any health issues, including sustaining the health behavior.

### Limitations and Future Research

This study measured intention to sustain a healthy behavior as a proxy of the actual behavior; however, actual behavior change requires longitudinal observations. In addition, the familiarity with technology tools construct did not focus on mHealth, but on general ICT; our implications for the design of mHealth apps are limited. It is also important to note that although this research was about empowering people to control and maintain their health, 79.9% (341/427) of the respondents did not have any chronic disease, and 57.4% (245/427) of the respondents assessed their health as very good or excellent (Table 2). Thus, it may well be that healthy persons do not perceive the necessity of being empowered (able to control their health) and sustaining healthy behavior as much as the less healthy persons. A survey conducted with healthy individuals may yield different results from a survey conducted with unhealthy individuals. For example, the healthy respondents (as they may not find keeping fit necessary for them at that stage of life) may not find the general goal of having regular exercise and experientially rewarding contents relevant to them as much as the unhealthy respondents would perceive. In addition, considering the respondents’ age, which is mostly below 40 years, a bias of answers on the familiarity with technology could be identified and induce a limitation. Thus, interpretation and generalization of our findings to the context of patient empowerment must be performed with careful consideration of these limitations. Future research should validate these findings from the perspectives of patients. Qualitative research could be conducted to develop a deeper and more holistic understanding of what could lead to the feeling of empowerment and how the feeling of empowerment lead to an intention of sustained health behavior. In addition, longitudinal research could allow us to observe whether the actual behavior will persist and allow a richer understanding of how to sustain health-protective behavior.

### Conclusions

To better understand sustaining health behavior, we developed and tested a theoretical model of empowerment and an intention to maintain a healthy behavior. Our findings indicated that messages aligned with personal goals, self-efficacy, social connection, and community support are enablers of empowerment related to health issues. The feeling of empowerment increases an individual’s intention to sustain a healthy behavior. This suggested that to promote long-term healthy behavior through the use of technological tools, one will have to integrate personal and social factors into the tools that will be used as health empowering tools.

## Abbreviations

AVE: average variance extracted
CFI: comparative fit index
ICT: information and communications technology
ITHBC: Integrated Theory of Health Behavior Change
mHealth: mobile health
RMSEA: root-mean-square error of approximation
